# T-cell responses following Natural Influenza Infection or Vaccination in Solid Organ Transplant Recipients

**DOI:** 10.1038/s41598-020-67172-6

**Published:** 2020-06-22

**Authors:** Arnaud G. L’Huillier, Victor H. Ferreira, Cedric Hirzel, Srinivas Nellimarla, Terrance Ku, Yoichiro Natori, Atul Humar, Deepali Kumar

**Affiliations:** 1 Division of Pediatric Infectious Diseases, Department of Pediatrics, University Hospitals of Geneva & University of Geneva Medical School, Geneva, Switzerland; 2 Multi-Organ Transplant Program, University Health Network, Toronto Ontario, Canada; 3 Department of Infectious Diseases, Inselspital, Bern University Hospital, University of Bern, Bern, Switzerland; 4 Sanofi-Genzyme, Waltham Massachusetts, USA; 5 Division of Infectious Disease, University of Miami Miller School of Medicine, Miami, Florida USA; 6 Miami Transplant Institute, Miami, Florida USA

**Keywords:** Immunology, Diseases, Medical research, Pathogenesis

## Abstract

Little is known about cell-mediated immune responses to natural influenza infection in solid organ transplant (SOT) patients. The aim of our study was to evaluate the CD4^+^ and CD8^+^ responses to influenza A and B infection in a cohort of SOT patients. We collected peripheral blood mononuclear cells at influenza diagnosis and four weeks later from 31 SOT patients during the 2017–2018 influenza season. Infection-elicited influenza-specific CD4^+^ and CD8^+^ T-cell responses were measured using flow cytometry and intracellular cytokine staining and compared to responses following influenza vaccine in SOT patients. Natural infection was associated with a significant increase in CD4^+^ T-cell responses. For example, polyfunctional cells increased from 21 to 782 and from 193 to 1436 cells per 10^6^ CD4^+^ T-cells among influenza A/H3N2 and B-infected patients (p = 0.006 and 0.004 respectively). Moreover, infection-elicited CD4^+^ responses were superior than vaccine-elicited responses for influenza A/H1N1 (931 vs 1; p = 0.026), A/H3N2 (647 vs 1; p = 0.041) and B (619 vs 1; p = 0.004). Natural influenza infection triggers a significant increase in CD4^+^ T-cell responses in SOT patients. Infection elicits significantly stronger CD4^+^ responses compared to the influenza vaccine and thereby likely elicits better protection against reinfection.

## Introduction

Solid organ transplant (SOT) recipients are at increased risk for morbidity and mortality following influenza virus infection because of lifelong immunosuppression required to avoid graft rejection^1,2^. The main preventive strategy against influenza is yearly immunization with the inactivated influenza vaccine, which is recommended for all SOT recipients^3,4^. Nevertheless, transplant recipients may develop influenza despite adequate immunization^1^, which in part may be related to inadequate vaccine immunogenicity, and the discordance between circulating influenza strains and those included in annual vaccine formulations^3,5–7^.

The adaptive immune system is conceptually divided into humoral and cell-mediated immunity (CMI). The humoral arm is responsible for production of neutralizing antibodies, support antibody-dependent cellular cytotoxicity, opsonization and complement fixation, whereas the cellular arm allows for direct killing of the pathogen and assists in the production of neutralizing antibodies^8^. Both arms are important in the immune response against natural influenza infection^8^, and it is unclear whether the development of cellular or humoral immunity is a better correlate of protection against subsequent infection. Most of the data available about the development of immunity against natural influenza infection are focused on the antibody responses. This is most likely related to the fact that measurement of humoral immunity is significantly less labour-intensive, there is standardization of assays and a better consensus on cut-off values required for protection. However, some studies in immunocompetent persons show that CMI may be a better correlate of protection against influenza than humoral immunity in vaccinated patients with poor immune responses^9,10^. Moreover, CMI seems to be important in recovery from influenza infection and in virus clearance, and possibly in preventing complications, rather than strictly prevent infection^11^. Organ transplant recipients are on T-cell suppressing therapies which may impact formation of CMI responses not only against vaccine but also against influenza infection. The aim of our study was therefore to evaluate the CD4 + and CD8 + T-cell responses during natural influenza infection in a cohort of SOT patients and to compare it to the T-cell responses elicited by the influenza vaccine in this population.

## Results

### Influenza infection

#### Demographics

Thirty-five patients were enrolled between December 2017 and May 2018. Four patients were subsequently excluded from analysis because of unavailable PBMCs either at diagnosis or at follow-up. Among the 31 patients who completed the study, median time between symptom onset and blood collection was 6 days (interquartile range [IQR] 4–11) and median time between diagnosis and blood collection was 3 days (IQR 2–4). Median time between acute and convalescent specimen collection was 38 days (IQR 29–50). One patient underwent re-transplantation between the two specimens collection. Patient demographics are detailed in Table 1. Fifteen patients were infected with Influenza A and 16 with influenza B (Table 2). All influenza-infected patients received a 5-day course of oseltamivir within a median of 6 days (IQR 4–11) after symptom onset (Table 2).

**Table 1:** Demographics and Immunosuppression Regimens of Transplant Recipients in the Influenza Infection and Vaccine Cohorts.

	Influenza infection (n = 31)	Influenza vaccine (n = 25)	p-value
**Demographics**			
Age, y, median (IQR)	54.8 (39.0–63.9)	59.0 (49.5–62.5)	0.239
Male sex	19 (61.3)	22 (88.0)	**0.034**
Time after transplant, y, median (IQR)	2.4 (0.2–4.9)	3.5 (1.0–7.5)	0.259
**Type of transplant**			
Kidney	13 (41.9)	6 (24.0)	**0.001**
Liver	0 (0)	5 (20.0)	
Lung	16 (51.6)	5 (20.0)	
Heart	2 (6.5)	4 (16.0)	
Combined	0 (0)	5 (20.0)	
**Immunosuppression**			
ATG in the last 3 months	4 (12.9)	1 (4.0)	0.367
Prednisone	31 (100)	19 (76.0)	**0.005**
daily dose (mg), median (IQR)	15.0 (7.5–20.0)	5.0 (1.3–5.0)	**<0.001**
Calcineurin inhibitor	31 (100)	31 (100)	—
Tacrolimus	16 (51.6)	16 (64.0)	0.352
trough (µg/l), median (IQR)	8.1 (5.9–12.5)	6.8 (4.4–8.9)	0.251
Cyclosporine	15 (48.4)	9 (36.0)	0.352
trough (µg/l), median (IQR)	206 (163–256)	145 (102–271)	0.283
Sirolimus	0 (0)	1 (4.0)	0.446
MMF	19 (61.3)	18 (72.0)	0.4
daily dose (mg), median (IQR)	1440 (720–1440)	1040 (720–1440)	0.169
Azathioprine	1 (3.2)	3 (12.0)	0.314

IQR: interquartile range; ATG: antithymocyte globulin; MMF: mycophenolate mofetil.

**Table 2:** Characteristics of Transplant Recipients With Influenza Infection.

	Patients (n = 31)
**Infection**	
**Influenza, n(%)**	
A/H1N1	2 (6.5)
A/H3N2	13 (41.9)
B	16 (51.6)
**Symptoms, n(%)**	
Fever	19 (61.3)
Sore throat	15 (48.4)
Cough	30 (96.8)
Myalgia	12 (38.7)
Dyspnea	22 (71.0)
Headache	14 (45.2)
Rhinorrhea	19 (61.3)
Nausea or vomiting	8 (25.8)
Diarrhea	8 (25.8)
Fatigue	5 (16.1)
**Management**	
Oseltamivir, n (%)	31 (100)
Median daily dose, mg (IQR)	75 (60–150)
Median duration, days (IQR)	5 (5–5)
Median time symptom onset and antivirals, days (IQR)	6 (4–11)
**Complications**	
Admission, n (%)	28 (90.3)
Median duration admission, days (IQR)	5 (2–10)
Pneumonia, n (%)	12 (38.7)
Death at 6 months*, n (%)	3 (9.7)
Rejection in the month prior or the 3 months after infection#, n (%)	2 (6.5)
**Influenza immunization, n (%)**	
Same season	18/27 (66.7)
Previous season	17/24 (70.8)
**Laboratory results**	
Median creatinine clearance, mL/mn (IQR)	54 (33–81)
Median leukocyte count, G/l (IQR)	4.6 (4.0–6.3)
Median neutrophil count, G/l (IQR)	3.6 (1.5–5.4)
Median lymphocyte count, G/l (IQR)	0.7 (0.4–1.1)

*Causes of death were: bleeding from fistula 3 months following influenza infection (n = 1), M.abscessus infection 4 months after influenza infection (n = 1) and chronic lung allograft dysfunction 4 months after influenza infection (n = 1)

^#^Grade 1–2 rejection in the month preceeding influenza infection (n = 1), Grade 2 rejection 3 weeks following influenza infection (n = 1)

IQR: interquartile range.

#### CD4^+^ and CD8^+^ T-cell immunity at influenza diagnosis

**Figure 1:**
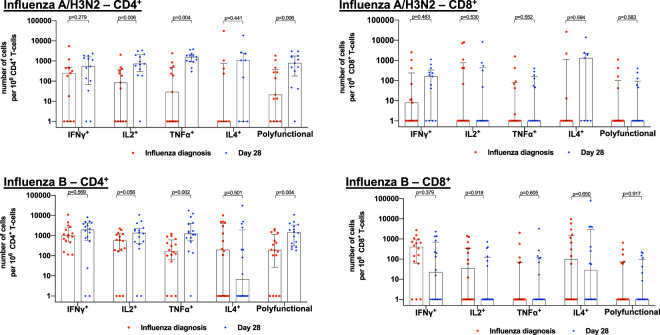
Evaluation of influenza-specific CD4^+^ and CD8^+^ T-cell immunity among influenza A/H3N2- and influenza B-infected patients at influenza diagnosis and Day 28. IFN-γ: interferon-γ; TNF-α: tumor-necrosis factor-α; IL: interleukin. Results were expressed as number of cytokine-producing CD4^+^ cells/10^6^ CD4^+^ T-cells or CD8^+^ cells/10^6^ CD8^+^ T-cells. For data presentation, cell frequencies labelled as TNFα^+^, IFNγ^+^, IL2^+^ or IL4^+^ were cells producing the given cytokine, regardless of the production of other cytokines. The upper edge of the box represents the median value, and the error bars represent the interquartile range. Red and blue dots represent individual patient frequencies at influenza diagnosis and Day 28, respectively.

Among patients infected with influenza A/H3N2, the median number of influenza A/H3N2-specific cytokine-producing T-cells at baseline ranged between 1-248 and 1–8 cells per 10^6^ CD4^+^ and CD8^+^ T-cells, respectively (Fig. 1 and Supplementary Table T1 online). Patients infected with influenza A/H1N1 (n = 2) had influenza A/H1N1-specific cytokine producing T-cells ranging between 1–28 and 1–41 cells per 10^6^ CD4^+^ and CD8^+^ T-cells, respectively (Supplementary Table T1 online). The median number of influenza B-specific cytokine producing T-cells at baseline ranged between 171–997 and 1-405 cells per 10^6^ CD4^+^ and CD8^+^ T-cells, respectively (Fig. 1 and Supplementary Table T1 online).

#### CD4^+^ and CD8^+^ T-cell immunity 28 days after influenza infection

Among patients infected with influenza A/H3N2, the median number of influenza A/H3N2-specific cytokine-producing CD4^+^ T-cells 28 days post-diagnosis ranged between 534–1577 cells per 10^6^ CD4^+^ T-cells. This represented a significant increase relative to the time of diagnosis for IL2^+^ (p = 0.006), TNFα^+^ (p = 0.004) and polyfunctional (p = 0.006) CD4^+^ T-cells (Fig. 1 and Supplementary Table T1 online). The median number of influenza A/H3N2-specific cytokine-producing CD8^+^ T-cells ranged between 1-1,312 cells per 10^6^ CD8^+^ T-cells. There was no significant increase in the number of influenza A/H3N2-specific cytokine-producing CD8^+^ T-cells at Day 28 when compared to baseline (Fig. 1 and Supplementary Table T1 online). Patients infected with influenza A/H1N1 (n = 2) had influenza A/H1N1-specific cytokine producing T-cells ranging between 1–1,739 and 1-959 cells per 10^6^ CD4^+^ and CD8^+^ T-cells, respectively (Supplementary Table T1 online). The median number of influenza B-specific cytokine-producing CD4^+^ T-cells ranged between 1-1,957 cells per 10^6^ CD4^+^ T-cells. This represented a significant increase when compared to diagnosis for TNFα^+^ (p = 0.002) and polyfunctional (p = 0.004) CD4^+^ T-cells (Fig. 1 and Supplementary Table T1 online). The median number of influenza B-specific cytokine-producing CD8^+^ T-cells ranged between 1–28 cells per 10^6^ CD8^+^ T-cells. There was no significant increase in the number of influenza B-specific cytokine-producing CD8^+^ T-cells at Day 28 when compared to the time of diagnosis (Fig. 1 and Supplementary Table T1 online).

Receipt of the TIV during the same influenza season did not impact the magnitude of the subsequent influenza-specific CD4^+^ T-cells response at influenza diagnosis or at Day 28 (Supplementary Fig. S1 online). Similarly, receipt of the TIV during the same influenza season did not impact the magnitude of the subsequent influenza-specific CD8^+^ T-cells response, with the exception of a stronger IL2^+^CD8^+^ T-cell response at influenza diagnosis among patients who received the TIV (p = 0.039; Supplementary Fig. S1 online).

The relative contribution of each cytokine to polyfunctional CD4^+^ and CD8^+^ T-cells in influenza A/H3N2- and B-infected patients is detailed in Supplementary Fig. S2 and S3 online. Interestingly, less than 20% of polyfunctional cells were triple-positive cells (Supplementary Fig. S2 and S3 online).

#### Analysis of heterosubtypic CD4^+^ and CD8^+^ T-cell immunity among influenza A-infected patients

**Figure 2:**
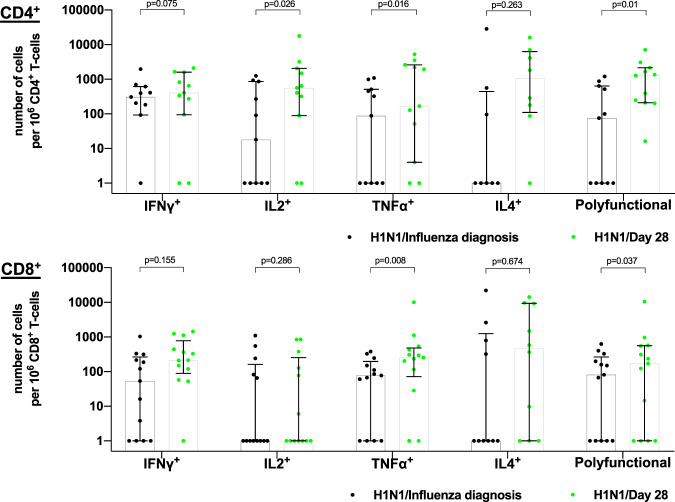
Evaluation of heterosubtypic influenza A/H1N1-specific CD4^+^ and CD8^+^ T-cell immunity among influenza A/H3N2-infected patients IFN-γ: interferon-γ; TNF-α: tumor-necrosis factor-α; IL: interleukin. Results were expressed as number of cytokine-producing CD4^+^ cells/10^6^ CD4^+^ T-cells or CD8^+^ cells/10^6^ CD8^+^ T-cells. For data presentation, cell frequencies labelled as TNFα^+^, IFNγ^+^, IL2^+^ or IL4^+^ were cells producing the given cytokine, regardless of the production of other cytokines. The upper edge of the box represents the median value, and the error bars represent the interquartile range. Black and green dots represent individual frequencies in influenza A/H3N2-infected patients at influenza diagnosis and Day 28, respectively.

Among influenza A/H3N2-infected patients, stimulation with H1N1 elicited a significant increase in IL2^+^, TNFα^+^ and polyfunctional CD4^+^ T-cells (Fig. 2), as seen with stimulation with H3N2 (Fig. 1) suggesting that natural infection does result in heterosubtypic immunity. Interestingly, in this group, stimulation with H1N1 also elicited a significant increase in TNFα^+^ and polyfunctional CD8^+^ T-cells (Fig. 2), which was not seen with stimulation with H3N2 (Fig. 1).

Among influenza A/H1N1-infected patients (n = 2), there was a CD4^+^ and CD8^**+**^ T-cell response to H3N2 stimulation at a similar level compared the response to H1N1 stimulation (data not shown).

#### Outcomes measures associated with polyfunctional CD4^+^ and CD8^+^ T-cells

There was a trend towards an association between protection from pneumonia or death and the presence of polyfunctional CD4^+^ T-cells at influenza diagnosis (83.3% [15/18] vs 53.8% [7/13]; p = 0.084). However, there was no association between protection from pneumonia or death and the presence of polyfunctional CD4^+^ T-cells at Day 28 (100% [18/18] vs 92.3% [12/13]; p = 0.419).

There was no association between protection against pneumonia or death and the presence of polyfunctional CD8^+^ T-cells, both at influenza diagnosis (38.9% [7/18] vs 38.5% [5/13]; p = 0.638) or at Day 28 (44.4% [8/18] vs 38.5% [5/13]; p = 0.516).

### Comparison of infection-elicited and vaccine-elicited responses

#### Demographics

We compared the T-cell responses elicited by influenza infection to the responses elicited by influenza vaccine in 25 SOT patients. There was no significant difference in age or time since SOT between the two groups (Table 1). There were significantly more lung transplant recipients in the influenza infection group. The influenza infection group was also more likely to be on prednisone and prednisone daily doses were higher (Table 1).

#### Cellular immunity

**Figure 3:**
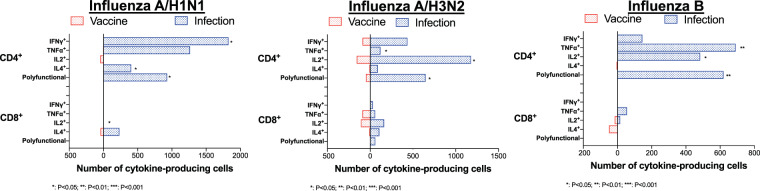
Comparison of median number of influenza-specific infection-elicited and vaccine-elicited cytokine-producing CD4^+^ and CD8^+^ T-cells in SOT recipients. SOT: Solid organ transplant; IFN-γ: interferon-γ; TNF-α: tumor-necrosis factor-α; IL: interleukin. Results were expressed as number of vaccine-elicited cytokine-producing CD4^+^ cells/10^6^ CD4^+^ T-cells and number of cytokine-producing CD8^+^ cells/10^6^ CD8^+^ T-cells. For data presentation, cell frequencies labelled as TNFα^+^, IFNγ^+^, IL2^+^ or IL4^+^ were cells producing the given cytokine, regardless of the production of other cytokines. The boxes represents the median value. Pre-vaccination frequencies were subtracted from post-vaccination frequencies to highlight vaccine-elicited responses, whereas frequencies at influenza diagnosis were subtracted from frequencies at Day 28 to highlight infection-elicited responses.

The proportion of infection-elicited IFNγ^+^, IL4^+^ and polyfunctional CD4^+^ T-cells among influenza A/H1N1-infected patients were significantly greater than vaccine-elicited CD4^+^ T-cells against influenza A/H1N1 (p = 0.033, 0.026 and 0.042 respectively) (Fig. 3 and Supplementary Fig. S4 online). Similarly, the proportion of infection-elicited TNFα^+^, IL2^+^ and polyfunctional CD4^+^ T-cells was significantly higher among influenza A/H3N2-infected (p = 0.038, 0.017 and 0.041 respectively) and influenza B-infected patients (p = 0.002, 0.048 and 0.004 respectively) than in vaccine recipients (Fig. 3 and Supplementary Fig. S4 online).

In general, the proportion of cytokine-producing CD8^+^ T-cells did not significantly differ between vaccinated and infected patients, with the exception of a higher frequency IL2^+^ CD8^+^ T-cells among influenza A/H1N1-infected patients when compared to vaccinated patients e (p = 0.021) (Fig. 3 and Supplementary Fig. S5 online).

## Discussion

We evaluated the dynamics of influenza-specific cell-mediated immunity in a cohort of SOT patients infected with influenza and compared T-cell responses to a cohort of vaccinated patients. Our study shows that influenza infection elicited T-cell responses primarily for CD4^+^. These responses also appear to be cross-reactive with other influenza strains (heterosubtypic response). We also showed that T-cell responses were significantly greater in infected patients compared to vaccinated patients. This was true not only for monofunctional cells, but also for polyfunctional cells, which may be a better correlate of the quality of the immune response. Our study provides novel comparative data on cellular immune response to natural vs. vaccine responses in transplant patients.

Upon infection, influenza virus activates innate immune components, leading eventually to the generation of influenza-specific CD8^+^ and CD4^+^ T-cell responses, both of which are important in controlling influenza infection^18^. CD8^+^ T-cells differentiate into cytotoxic T lymphocytes which control viral replication and kill infected cells through production of cytokines and effector molecules^12^. On the other hand, CD4 + T-cells activate B-cells to produce antibodies, but also target infected-cells^12^. There are limited data in transplant recipients looking at short-term T-cell response to seasonal influenza infection. Our group previously looked at long-term T-cell responses to influenza A/H1N1pdm in a cohort of 22 transplant recipients that had been infected with influenza during the 2009 influenza pandemic. At a median of 7 months post-infection, influenza-specific CD4 + and CD8 + T-cells responses were found in 50% and 36%, respectively^13^. This is consistent with the current study which also shows a predominantly CD4 + T-cell response. We also showed that both influenza A and B infections elicited a significant increase in polyfunctional CD4 + T-cells, which are considered to be a good correlate of the quality of the T-cell response^14^.

Several studies have demonstrated the importance of polyfunctional CD4 + T-cells in the control of influenza in non-transplant patients. Polyfunctional influenza-specific CD4^+^ cells have been shown to be functionally superior, as shown by increased degranulation and higher production of CD40L and cytokine per cell^15^. In a cohort of pregnant women infected with influenza A/H1N1pdm, the proportion of polyfunctional CD4 + T-cells was inversely correlated with influenza disease severity^16^, confirming murine data where polyfunctional cells also protected against lethal influenza infection^17^. Similarly, polyfunctional cells have been associated with better protection after vaccination and better outcomes in several other models^18,19^. In our cohort, there was a trend towards an association between the presence of polyfunctional CD4 + T-cells at influenza diagnosis and reduced disease severity. The development of CMI is especially important in older adults age ≥65 years who are known to derive poor protection from vaccination. In this group, CMI may be a better correlate of protection than antibody response^9,10^. We postulate that CMI likely has a major role to play in SOT patients as well.

In our current infected cohort, 67% had received the influenza vaccine prior to being infected. Interestingly, there was no difference in the T-cell responses at influenza diagnosis or at Day 28 in vaccinated vs. unvaccinated patients. This may be due to the size of the cohort or the lack of sustained T-cell responses following the vaccine.

We also showed evidence that transplant patients can develop heterosubtypic immunity as evidenced by response to H1N1 stimulation in H3N2-infected patients. The heterosubtypic response may be mediated by T-cell immunity directed against conserved influenza antigens^20^. We did not find that infection elicited a significant homotypic or heterosubtypic CD8^+^ T-cell response. CD8^+^ T-cell responses are important in cytotoxicity and are the basis of some CD8^+^ directed universal influenza vaccines. There are several possible explanations for the low CD8^+^ responses observed in our study. First, the antigen used for stimulation was calibrated on HA concentration, which is known to predominantly stimulate CD4^+^ responses whereas internal influenza proteins such as M1 and NP predominantly stimulate CD8^+^ responses^21^. The use of inactivated virus calibrated on HA concentration could potentially have underestimated CD8^+^ responses, even though it is not possible to know whether the other antigens – including internal proteins - were in lower, similar or higher respective concentration than HA. Another reason for lower CD8^+^ responses may be that we did not use a more sensitive technique such as EliSPOT or Fluorospot. Finally, the low CD8^+^ responses might be related to the iatrogenic immunosuppression in our cohort that could affect CD8^+^ responses to a greater extent than CD4 + responses; this is supported by data showing stronger CD4^+^ than CD8^+^ responses against pandemic H1N1 in SOT patients while using live virus for stimulation^13^. Therefore, even if SOT patients may mount significant CD8^+^ responses, we did not demonstrate these in the current study. It is interesting that we have previously found polyfunctional CD8^+^ T-cell responses in SOT patients who were vaccinated with high-dose TIV^22^ but less so with natural infection. There may be a number of reasons for this. Unlike vaccination, infection may generate CD8 + immune-escaped variants in immunocompromised patients^23^. Vaccination also occurs intramuscularly and in high antigenic doses which may skew responses. On the other hand, infection is mucosal and the immune response may be directed against different epitopes. Therefore, the antigens used to re-stimulate the response in an experimental setting may not be the same as the response against the circulating strain.

One of the main findings of our study is that CD4^+^ responses in infected patients were significantly greater than those in noninfected, vaccinated patients. This finding occurred despite some important differences between the two cohorts that would suggest the infected cohort might have had poorer immune responses. The infected cohort had significantly more lung transplant recipients and more patients receiving prednisone along with higher daily doses of prednisone. Despite the potential for greater immunosuppression in the infected group, T-cell responses were greater. In general, natural infection should elicit a greater T-cell response than vaccination. For example, in healthy adults, the influenza vaccine is associated with lower antibody titers and rapidly waning antibodies when compared to natural influenza infection^24,25^. Prior data in the general population has shown that CMI responses to influenza vaccine are diminished compared to CMI generated with natural infection^26,27^. Interestingly, in our study, the influenza vaccine elicited a predominant IL2 + CD4 + response to H1N1 whereas influenza infection elicited a predominant IFNγ + CD4 + response, which is in line with previous data^28^. Interestingly, the high-dose split-virus influenza vaccine (FluzoneHD, Sanofi, Canada) elicited CD4^+^ and CD8^+^ T-cell responses at a similar level compared to influenza infection, using the same flow cytometry parameters^22^.

This study has some limitations. Our sample size was relatively small, even though we were still able to show a significant increase in CMI after infection and significant differences in CMI when compared to patients from the vaccine study. The sample size however did not allow to define correlates of protection against severe outcomes. There were only two A/H1N1-infected patients in our cohort likely because the local predominant circulating strains were A/H3N2 and B in respectively 46% and 50% of cases during the 2017–2018 season^29^. Serum was not collected, which did not allow to evaluate whether there was a correlation between humoral and cellular responses. We also did not examine heterosubtypic responses to other influenza B strains. Influenza infected patients were enrolled in a different season than vaccinated patients and this may have introduced variability into the results. However, our cell stimulations were based on antigens corresponding to circulating wild strains and strains included in the vaccine formulation. There was an enrollment bias towards admitted patients. Moreover, median time between symptom onset and blood collection was six days, which could have biased our interpretation of T-cell responses at influenza diagnosis infection onset because of an already ongoing immune response. However, the bias would underestimate the infection-elicited responses as well as differences between infection-elicited and vaccine-elicited responses, which would further strengthen our findings. Our study did not define which influenza proteins/epitopes were responsible for the effector responses and this could be done in future studies. Similarly, the fact that we used inactivated viruses quantified on HA concentration and not internal proteins might have biased the responses towards CD4 and therefore does not allow strong conclusions about CD8 + responses. Finally, although this was not the primary goal of our study, comparing our data with a control group of healthy adults infected with influenza would also allow further insights.

In conclusion, our study provides novel data on the cellular immune response to influenza in SOT patients, and shows that natural influenza infection elicited a significant increase in monofunctional and polyfunctional influenza-specific CD4^+^ T-cells. Interestingly, among patients infected with influenza A, heterosubtypic and homotypic stimulation elicited a similar T-cell response, suggesting potential cross-protective immunity. Finally, the level of the T-cell response following natural influenza infection was significantly higher than the response following the TIV. More studies are required to evaluate how this translates in term of protection against (re)infection.

## Methods

### Study design

Adult organ transplant recipients at the Toronto Transplant Program were enrolled if they had microbiologically confirmed influenza infection during the 2017–2018 influenza season. Whole blood for peripheral blood mononuclear cells (PBMC) isolation was collected at diagnosis and 4 weeks post-diagnosis. The study was approved by the University Health Network research ethics board and written consent was obtained from all patients before data and specimen collection. Detailed clinical information was gathered and patients were followed for six months following influenza diagnosis. Vaccinated patients were also adult organ transplant recipients and were enrolled as part of a randomized trial of influenza vaccination conducted during the 2016–2017 influenza season^30^. For purposes of the current study, only patients that received standard split-virus trivalent influenza vaccine (TIV) (Fluviral, GSK, Canada) and provided PBMCs in that trial were used as a comparison group. All methods were performed in accordance with the relevant guidelines and regulations, including the Declaration of Helsinki. No organ or tissue was procured from prisoners.

### Influenza diagnosis and subtyping

Influenza was diagnosed using the Luminex Aries Flu A/B & RSV PCR assay (Luminex Corporation, Austin, TX, USA) on nasopharyngeal (NP) swab or broncho-alveolar lavage (BAL) specimens. Influenza A was subtyped at the Public Health Ontario Laboratories (PHOL) using an in-house real-time PCR for which primer and probe sequences were based on Centers for Disease Control (CDC) references and target HA surface protein^31^.

### PBMC Stimulation and Flow Cytometry

For influenza infected patients, whole blood was obtained at influenza diagnosis and 28 days later. PBMCs were immediately isolated from blood using Ficoll (GE Healthcare Life Science, Issaquah, WA, USA) gradient centrifugation and cryopreserved in fetal bovine serum (ThermoFisher Scientific, Waltham, MA, USA) with 10% dimethyl sulfoxide (Fisher BioReagents, ThermoFisher Scientific), as previously described^22^. To determine influenza-specific T-cell responses 5×10^5^ PBMCs were stimulated immediately after thawing with 1 µg/ml of either influenza antigens corresponding to circulating seasonal H1N1, H3N2 or B viruses or media alone for 16 hours. The anti-human CD28/CD49d co-stimulatory reagent (BD FastImmune^TM^, BD Biosciences, Mississauga, ON, Canada) and protein transport inhibitor (eBioscience, ThermoFisher Scientific) were also added immediately after thawing, as previously described^22^. Viral antigens corresponding to A/Michigan/45/2015 (H1N1), A/Hong Kong/4801/2014 (H3N2) and B/Phuket/3073/2013 were obtained from the National Institute for Biological Standards and Control (NIBSC, Hertfordshire, UK) and reconstituted according to the manufacturer’s instructions (Influenza Antigens A/Michigan/45/2015 [NYMC X-275; NIBSC code 17/154], A/Hong Kong/4801/2014 [NYMCX-263B; NIBSC code 16/286] and B/Phuket/3073/2013 [NIBSC code 16/168], respectively). These antigens corresponded to respectively 100%, 100% and 98.6% of H1N1, H3N2 and B strains typed in Ontario, Canada during the 2017–2018 influenza season in which patients were enrolled^29^. For influenza B-infected patients, only homotypic responses were evaluated whereas homotypic and heterosubtypic responses were evaluated for influenza A-infected patients. Heterosubtypic responses were defined as a T-cell response to a different influenza subtype than the subtype which infected the patient (eg, T-cell response to influenza A/H1N1 for patients infected with A/H3N2).

For patients who received the vaccine, PBMCs were obtained pre-vaccination and 4 weeks post-vaccination and were stimulated with the viral antigens that corresponded to the antigens in the 2016–2017 trivalent vaccine and obtained from the NIBSC, as previously described^22^.

Following stimulation, cells were spun down and stained with the Zombie NIR^TM^ viability dye (BioLegend, San Diego, CA, USA). Following Fc receptor blocking using Human BD Fc Block (BD Biosciences), cells were incubated with a cell-surface cocktail consisting of [mouse] anti-human CD45 (clone HI30)-PerCP/Cy5.5 (Biolegend), [mouse] anti-human CD3 (clone OKT3)-PE/Cy7 (Biolegend), [mouse] anti-human CD4 (clone SK3)-Pacific Blue (Biolegend), [mouse] and anti-human CD8 (clone RPA-T8)-V500 (BD Biosciences), as previously described^22^. Following incubation with fixation buffer (BioLegend) cells were treated with an intracellular cytokine antibody cocktail prepared in Intracellular Staining Permeabilization Wash Buffer (BioLegend)^22^. The intracellular cocktail consisted of [mouse] anti-human interferon (IFN)-γ (clone 4 S.B3)-FITC (Biolegend), [mouse] anti-human tumor-necrosis factor (TNF)-α (clone MAb11)-BV605 (Biolegend), [rat] anti-human interleukin (IL)-2 (clone MQ1–17H12)-BV650 (Biolegend), and [rat] anti-human IL-4 (clone MP4–25D2)-PE-Dazzle (Biolegend), as previously described^22^. Flow cytometry was performed on a BD LSR II at The SickKids-UHN Flow and Mass Cytometry Facility (Toronto, ON, Canada) with a target event count of 100,000 live, CD45^+^ cells. The data acquired was analysed using FlowJo software v10 (FlowJo LLC, Ashland, OR, USA). To account for background cytokine production, the frequency of cytokine-production obtained in the unstimulated specimen was subtracted from the frequency obtained in the stimulated specimen. Furthermore, to compare infection-elicited and vaccine-elicited responses, the frequencies of cytokine-producing cells at influenza diagnosis were subtracted from the frequencies at Day 28, whereas pre-vaccination frequencies were subtracted from the frequency post-vaccination. In the absence of vaccine-associated response, specimens were given a value of 1 cytokine-producing cell per 10^6^ CD4^+^ or CD8^+^ T-cells. For data presentation, cell frequencies labelled as TNFα^+^, IFNγ^+^, IL2^+^ or IL4^+^ were cells producing the given cytokine, regardless of the production of other cytokines. Polyfunctional cells were defined as cells producing two or more cytokines out of IFN-γ, TNF-α and IL-2 as determined using Boolean gating. We did not consider IL-4 as part of polyfunctionality based on previous published data that focuses primarily on TNF-α, IFN-γ, and IL-2, which are a typical Th1 subset^18,19^. However, given the importance of IL-4 as a hallmark Th2 cytokine, IL-4 was evaluated as an individual cytokine along with TNF-α, IFN-γ, and IL-2. A representative gating strategy for the identification of cytokine-producing CD4^+^ and CD8^+^ T-cells is presented in Supplementary Fig. S6 online. Cell counts of cytokine-producing cells were expressed per 10^6^ CD4^+^ or CD8^+^ T-cells.

### Statistical analysis

Analyses were performed in infected patients only for which specimens were available both at influenza diagnosis and at Day 28 (per-protocol analysis). For group comparisons, Chi-squared or Fisher exact test were used for categorical variables, whereas Mann-Whitney U test was used for continuous unrelated variables and Wilcoxon signed rank test was used for continuous, paired variables. A composite outcome of pneumonia and death was chosen as a marker of disease severity and used to evaluate whether the presence of polyfunctional cells (defined here as polyfunctional cells in frequencies greater than 0% of the parent gate once normalised to the background control) protected against adverse outcomes. Statistics were performed using SPSS version 23.0 (IBM Corp., Armonk, NY, USA). Figures were made using GraphPad 7.0 (LaJolla, CA, USA).

## Supplementary information

Supplementary information.
