# Supplementary material for: T-cell responses following Natural Influenza Infection or Vaccination in Solid Organ Transplant Recipients
Source: Sci Rep. 2020 Jun 22;10:10104. doi: 10.1038/s41598-020-67172-6 (PMC7308384; doi:10.1038/s41598-020-67172-6)
Supplement: Supplementary file 1 — Supplementary information. [file 41598_2020_67172_MOESM1_ESM.pdf]

T-cell responses following Natural Influenza Infection or Vaccination in Solid Organ Transplant Recipients

Arnaud G. L'Huillier[1,2], Victor H. Ferreira[2], Cedric Hirzel[2,3], Srinivas Nellimarla[4], Terrance Ku[2], Yoichiro Natori[5,6], Atul Humar[2*] and Deepali Kumar[2*]

*Joint senior authorship

[1]Division of Pediatric Infectious Diseases, Department of Pediatrics, University Hospitals of Geneva & University of Geneva Medical School, Geneva, Switzerland.

[2]Multi-Organ Transplant Program, University Health Network, Toronto, Ontario, Canada.

[3]Department of Infectious Diseases, Inselspital, Bern University Hospital, University of Bern, Bern, Switzerland.

[4]Sanofi-Genzyme, Waltham, Massachusetts, USA

[5]Division of Infectious Disease, University of Miami Miller School of Medicine, Miami, Florida, USA.

[6]Miami Transplant Institute, Miami, Florida, USA.

Supplementary Figure 1. Comparison of CD4⁺ and CD8⁺ T-cell responses among influenza A/H3N2- and influenza B-infected patients based on the influenza vaccine status in the same season.

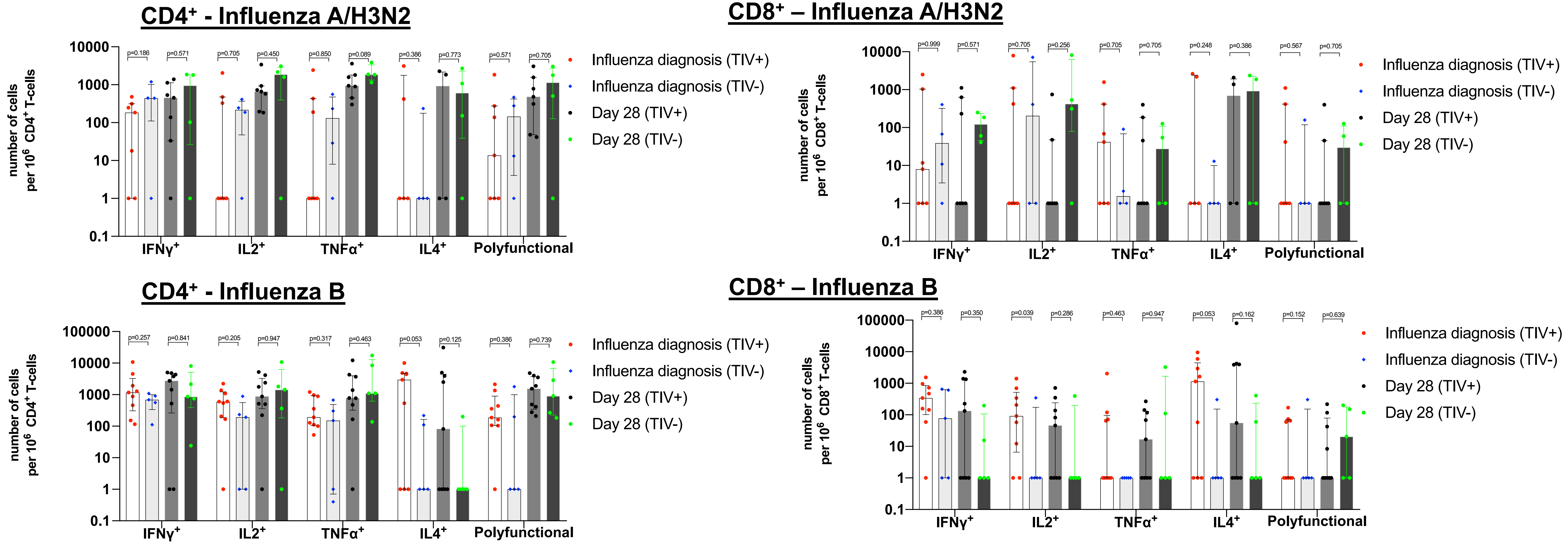

IFN-γ: interferon-γ; TNF-α: tumor-necrosis factor-α; IL: interleukin; TIV: trivalent inactived influenza vaccine. Results were expressed as number of cytokine-producing CD8⁺ cells/10⁶ CD8⁺ T-cells. For data presentation, cell frequencies labelled as TNFα⁺, IFNγ⁺, IL2⁺ or IL4⁺ were cells producing the given cytokine, regardless of the production of other cytokines. The upper edge of the box represents the median value, and the error bars represent the interquartile range

Supplementary Figure 2. Relative contribution of IFN-γ, TNF-α and IL-2 to polyfunctional CD4$^+$ and CD8$^+$ T-cells among the influenza A/H3N2-infected patients.

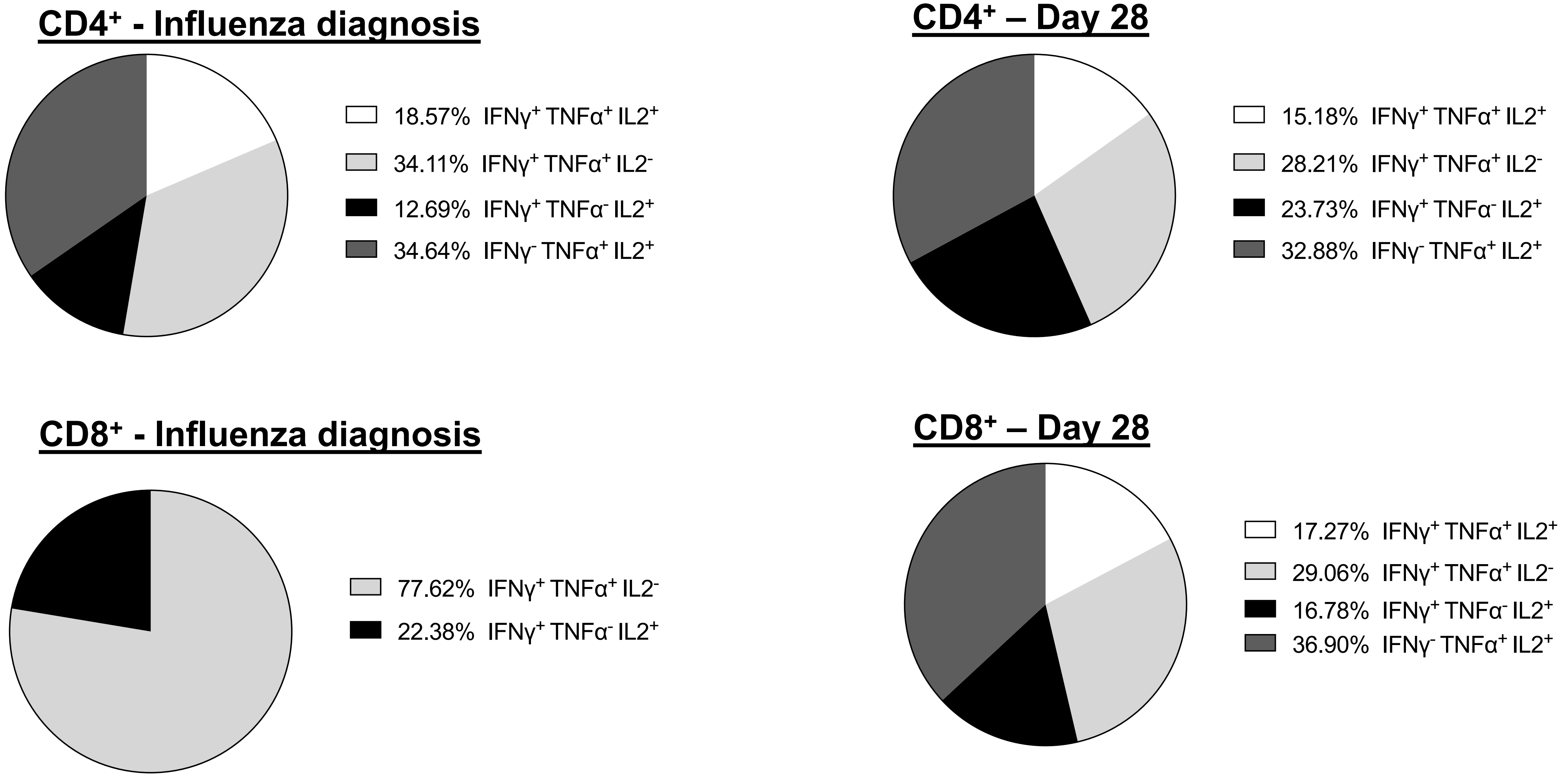

IFN-γ: interferon-γ; TNF-α: tumor-necrosis factor-α; IL: interleukin.
Data represent mean contribution of each cytokine to polyfunctional CD4$^+$ and CD8$^+$ T-cells.

Supplementary Figure 3. Relative contribution of IFN-γ, TNF-α and IL-2 to polyfunctional CD4$^+$ and CD8$^+$ T-cells among the influenza B-infected patients.

CD4$^+$ - onset

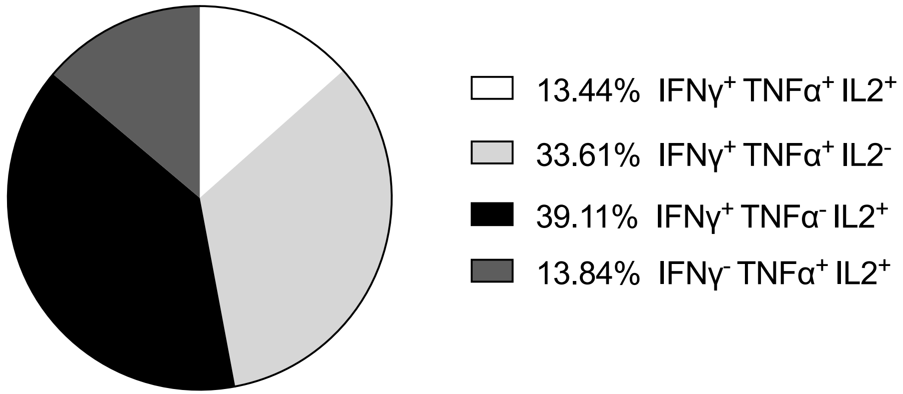

CD4$^+$ – Day 28

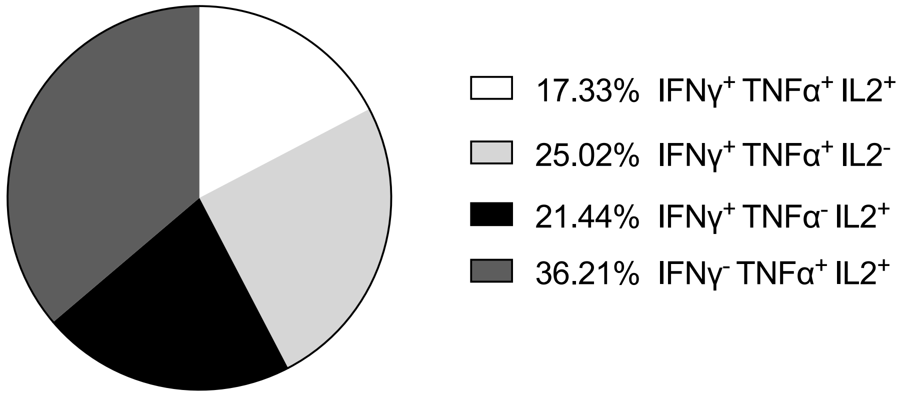

CD8$^+$ - onset

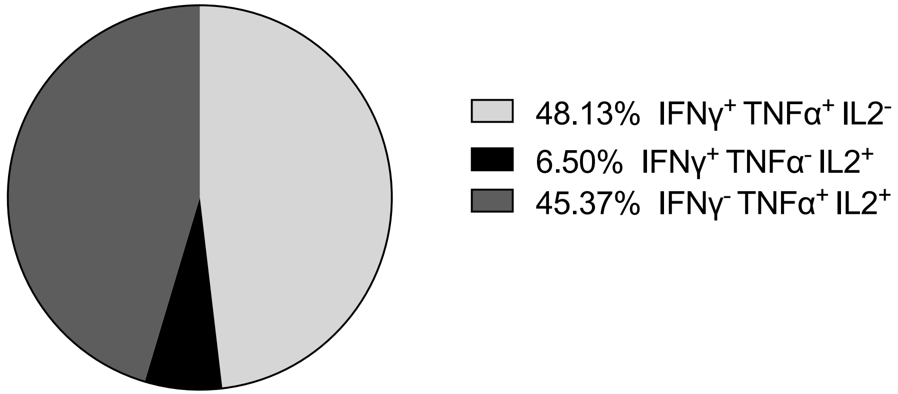

CD8$^+$ – Day 28

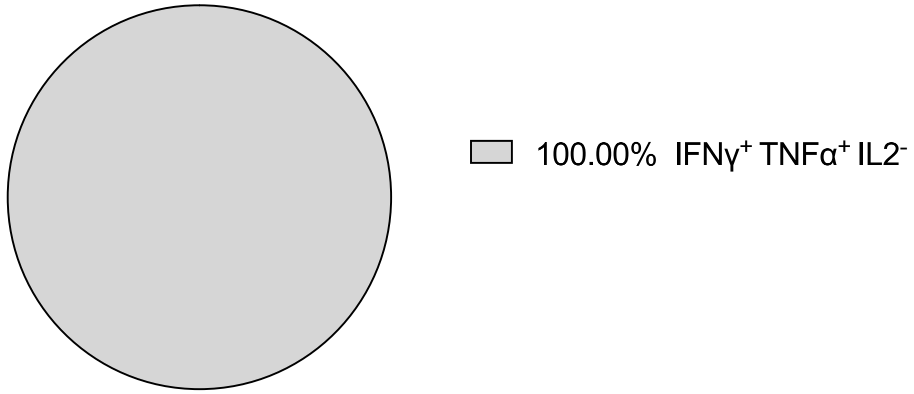

IFN-γ: interferon-γ; TNF-α: tumor-necrosis factor-α; IL: interleukin.
Data represent mean contribution of each cytokine to polyfunctional CD4$^+$ and CD8$^+$ T-cells.

Supplementary Figure 4. Comparison of median number of vaccine-elicited or infection-elicited influenza-specific cytokine-producing CD4⁺ T-cells after stimulation with influenza A/H1N1, influenza A/H3N2 and influenza B.

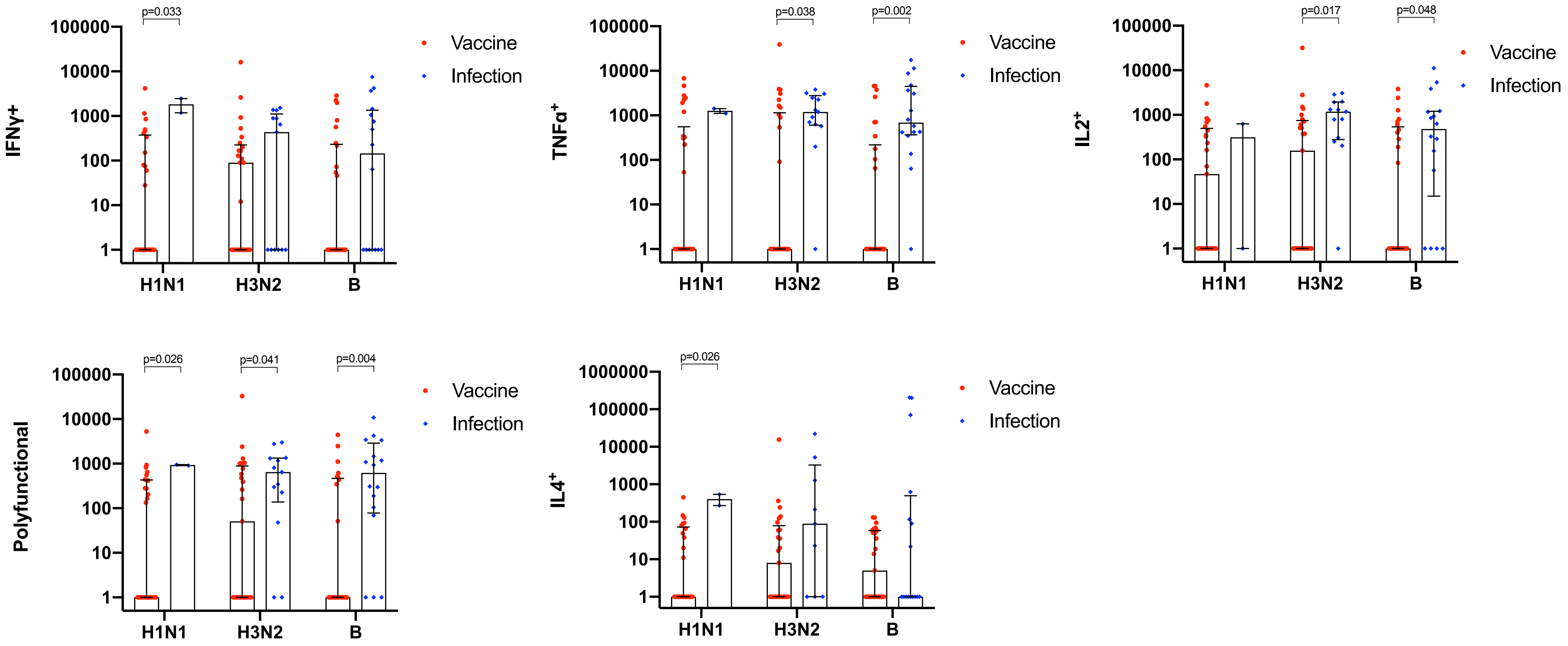

IFN-γ: interferon-γ; TNF-α: tumor-necrosis factor-α : IL: interleukin
Results were expressed as number of cytokine-producing CD4⁺ cells/10^6 CD4⁺ T-cells. Pre-vaccination frequencies were subtracted from post-vaccination frequencies to highlight vaccine-elicited responses, whereas frequencies at infection onset were subtracted from frequencies at day 28 to highlight infection-elicited responses. For data presentation, cell frequencies labelled as TNFα⁺, IFNγ⁺, IL2⁺ or IL4⁺ were cells producing the given cytokine, regardless of the production of other cytokines. The upper edge of the box represents the median value, and the error bars represent the interquartile range.

Supplementary Figure 5. Comparison of median number of vaccine-elicited or infection-elicited influenza-specific cytokine-producing CD8⁺ T-cells after stimulation with influenza A/H1N1, influenza A/H3N2 and influenza B.

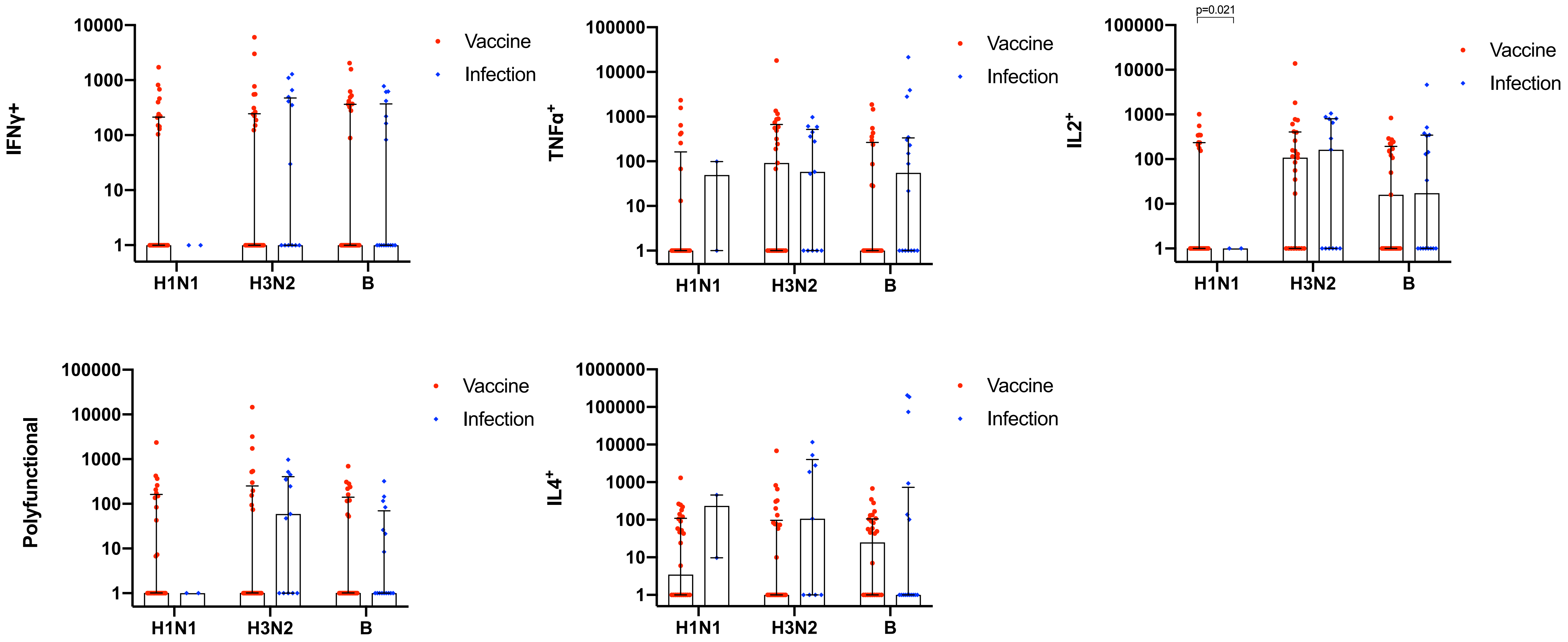

IFN-γ: interferon-γ; TNF-α: tumor-necrosis factor-α : IL: interleukin

Results were expressed as number of cytokine-producing $CD8^+$ cells/10^6 $CD8^+$ T-cells. Pre-vaccination frequencies were subtracted from post-vaccination frequencies to highlight vaccine-elicited responses, whereas frequencies at infection onset were subtracted from frequencies at day 28 to highlight infection-elicited responses. For data presentation, cell frequencies labelled as $TNF\alpha^+$, $IFN\gamma^+$, $IL2^+$ or $IL4^+$ were cells producing the given cytokine, regardless of the production of other cytokines. The upper edge of the box represents the median value, and the error bars represent the interquartile range.

Supplementary Figure 6. Representative flow cytometry gating strategy

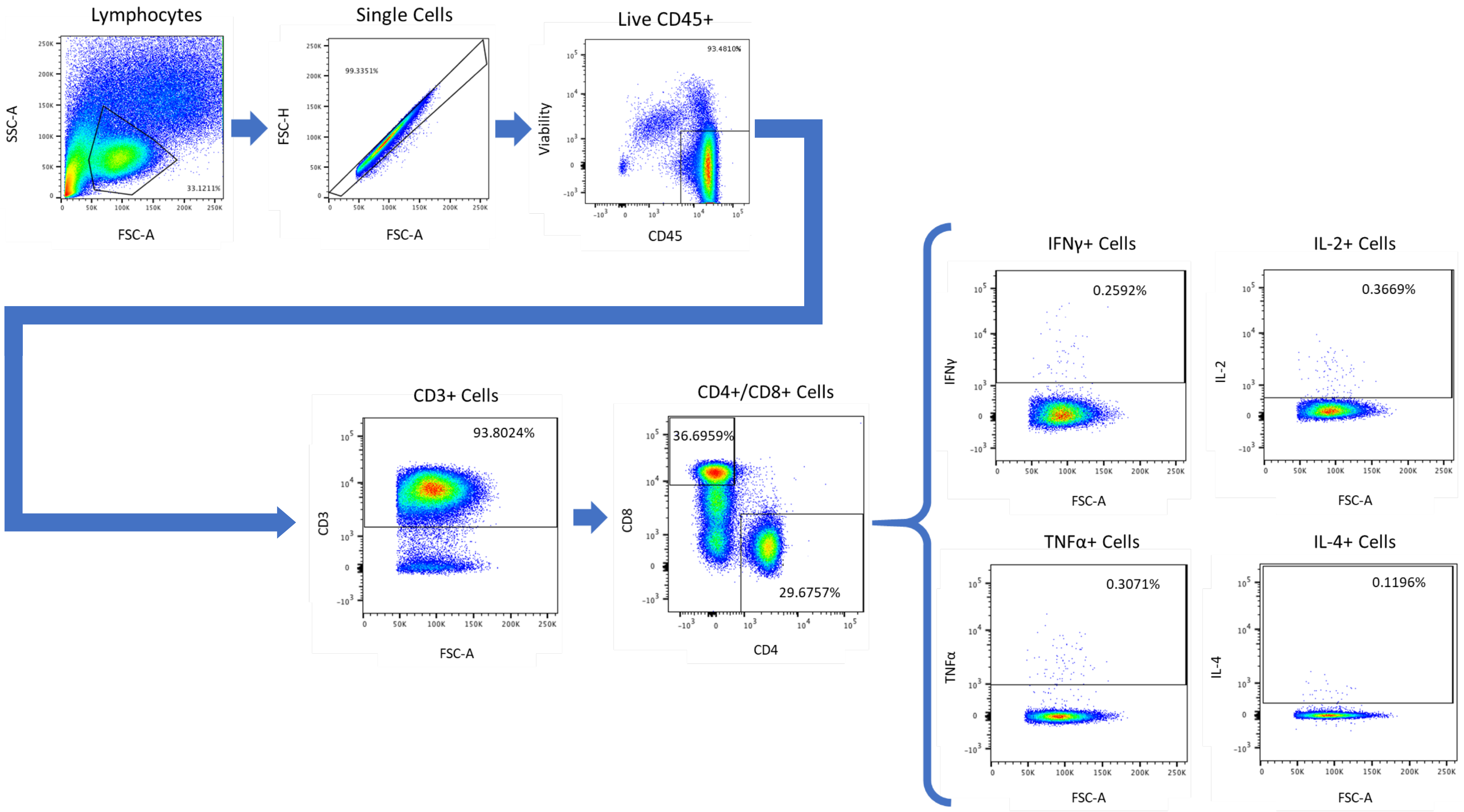

Supplementary Table 1. $CD4^{+}$ and $CD8^{+}$ T-cell immunity at influenza diagnosis and Day 28

	**A/H1N1-infected patients (n=2)**		**A/H3N2-infected patients (n=13)**		**B-infected patients (n=16)**	
	**Diagnosis** Median (IQR)	**Day 28** Median (IQR)	**Diagnosis** Median (IQR)	**Day 28** Median (IQR)	**Diagnosis** Median (IQR)	**Day 28** Median (IQR)
**$CD4^{+}$**						
$IFN\gamma^{+}$	- (1-1)	- (998-1734)	248 (1-462)	534 (68-1603)	997 (486-2525)	1957 (580-4218)
$TNF\alpha^{+}$	- (1-1)	- (1081-1124)	29 (1-494)	1577 (917-2093)	171 (64-612)	1246 (550-4125)
$IL2^{+}$	- (1-1)	- (1-1)	84 (1-369)	727 (293-2061)	570 (172-1180)	1358 (397-2304)
$IL4^{+}$	- (1-1)	- (253-299)	1 (1-742)	1071 (1-2484)	196 (1-4053)	1 (1-193)
Polyfunctional	- (1-28)	- (910-959)	21 (1-372)	782 (180-1744)	193 (22-1162)	1436 (311-3358)
**$CD8^{+}$**						
$IFN\gamma^{+}$	- (4-168)	- (1-123)	8 (1-236)	159 (1-331)	405 (64-903)	22 (1-685)
$TNF\alpha^{+}$	- (1-1)	- (1-1)	1 (1-79)	1 (1-157)	1 (1-70)	1 (1-115)
$IL2^{+}$	- (66-308)	- (1-1)	1 (1-761)	1 (1-424)	35 (1-342)	1 (1-123)
$IL4^{+}$	- (1-1)	- (10-188)	1 (1-1095)	1312 (1-2225)	99 (1-1504)	28 (1-2997)
Polyfunctional	- (1-41)	- (1-1)	1 (1-99)	1 (1-92)	1 (1-72)	1 (1-98)

IFN-γ: interferon-γ; TNF-α: tumor-necrosis factor-α : IL: interleukin; IQR: interquartile range

Results were expressed as number of cytokine-producing $CD8^{+}$ cells/10° $CD8^{+}$ T-cells. For data presentation, cell frequencies labelled as $TNF\alpha^{+}$, $IFN\gamma^{+}$, $IL2^{+}$ or $IL4^{+}$ were cells producing the given cytokine, regardless of the production of other cytokines.
